# The emerging modulators of non-coding RNAs in diabetic wound healing

**DOI:** 10.3389/fendo.2024.1465975

**Published:** 2024-10-08

**Authors:** Sis Aghayants, Jinjin Zhu, Jing Yu, Rui Tao, Sicheng Li, Shengzhi Zhou, Yunhua Zhou, Zhanyong Zhu

**Affiliations:** ^1^ Department of Plastic Surgery, Renmin Hospital of Wuhan University, Wuhan, Hubei, China; ^2^ Department of Dermatology, Union Hospital, Tongji Medical College, Huazhong University of Science and Technology (HUST), Wuhan, China; ^3^ Department of Plastic and Cosmetic Surgery, Tongji Hospital, Tongji Medical College, Huazhong University of Science and Technology, Wuhan, China; ^4^ Department of Wound Repair Surgery, Liyuan Hospital, Tongji Medical College, Huazhong University of Science and Technology, Wuhan, Hubei, China

**Keywords:** diabetic wound, diabetic foot ulcer, non-coding RNA, microRNA, long non-coding RNA, circular RNA

## Abstract

Diabetic wound healing is a complex physiological process often hindered by the underlying metabolic dysfunctions associated with diabetes. Despite existing treatments, there remains a critical need to explore innovative therapeutic strategies to improve patient outcomes. This article comprehensively examines the roles of non-coding RNAs (ncRNAs), specifically microRNAs (miRNAs), long non-coding RNAs (lncRNAs), and circular RNAs (circRNAs), in regulating key phases of the wound healing process: inflammation, angiogenesis, re-epithelialization, and tissue remodeling. Through a deep review of current literature, we discuss recent discoveries of ncRNAs that have been shown to either promote or impair the wound healing process in diabetic wound healing, which were not covered in earlier reviews. This review highlights the specific mechanisms by which these ncRNAs impact cellular behaviors and pathways critical to each healing stage. Our findings indicate that understanding these recently identified ncRNAs provides new insights into their potential roles in diabetic wound healing, thereby contributing valuable knowledge for future research directions in this field.

## 1 Introduction

**Figure 1:**
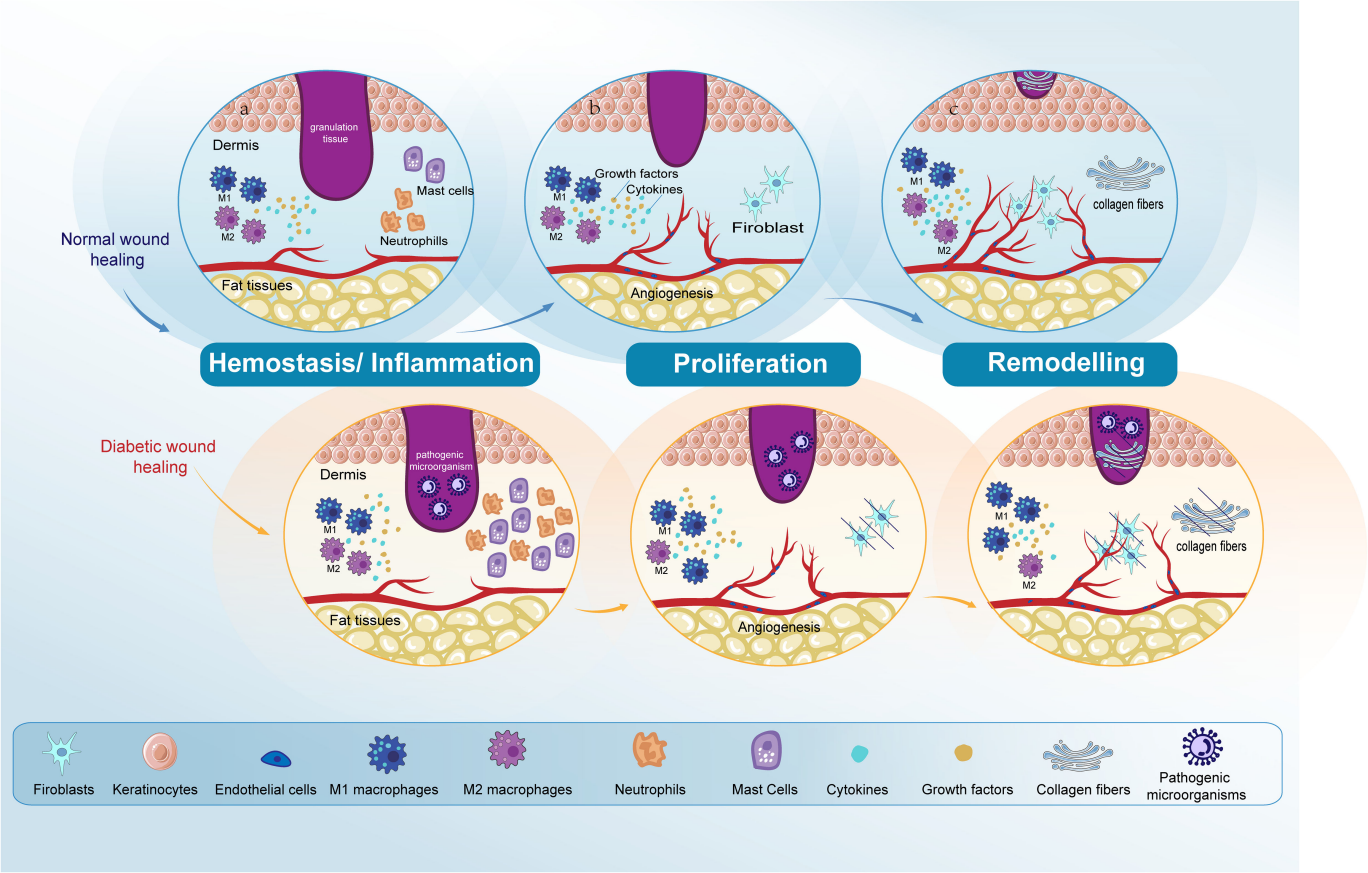
Comparative analysis of normal and diabetic wound healing phases. The figure compares normal wound healing with diabetic wound healing in the Hemostasis/Inflammation, Proliferation, and Remodeling phases. **(A)** Hemostasis/Inflammation phase: In normal wound healing, this phase is characterized by a balanced inflammatory response. In contrast, diabetic wound healing shows an increased presence of neutrophils and mast cells. M1 macrophage polarization is observed in diabetic wounds, leading to prolonged inflammation and impaired healing. **(B)** Proliferation phase: In normal wound healing, keratinocytes and fibroblasts proliferate and migrate effectively, and angiogenesis progresses normally. However, in diabetic wounds, there is impaired proliferation and migration of keratinocytes and fibroblasts. Angiogenesis is also impaired due to dysfunctional ECs. **(C)** Remodeling phase: In normal wound healing, this phase results in the final formation of the ECM and the completion of angiogenesis. Conversely, in diabetic wound healing, both ECM formation and angiogenesis remain impaired. Endothelial cells (ECs); extracellular matrix (ECM).

Skin repair is a tightly regulated biological procedure, comprising: hemostasis, inflammation, proliferation, and remodeling (1, 2). Hemostasis initiates when the vascular endothelium is damaged, leading to the formation of a platelet plug and a fibrin clot to stop bleeding, a process that takes minutes. The inflammatory phase starts right after hemostasis, this phase begins with an influx of neutrophils, followed by the arrival of monocytes that differentiate into macrophages within the tissues to clear away any remaining cellular remnants and expended neutrophils, lasting between three to five days (3, 4). In the next proliferation phase, the process encompasses the generation of new stromal structures by fibroblasts, the beginning of angiogenesis, extracellular matrix (ECM) formation, and collagen synthesis (5). During the final remodeling phase, the structure of the tissue is refined through the final formation of the ECM and further neovascularization. This phase sees significant contributions from fibroblasts, which produce fibroblast growth factors (FGF), and from vascular endothelial cells (ECs). The remodeling process, pivotal for restoring tissue integrity, can last up to two years (6–8) (**Figure 1**).

In comparison to acute wounds, chronic wounds often exhibit pathological irregularities and impairments in different cell functions, such as those of neutrophils, macrophages, ECs, fibroblasts, and keratinocytes (6, 9). Non-healing wounds pose a major challenge to public health (10). Diabetic skin exhibits increased infiltration of inflammatory cells and reduced granulation tissue formation compared to normal skin. The primary factors that complicate diabetic wound healing, in contrast to normal wound healing, include elevated glucose levels, hypoxia, and high levels of reactive oxygen species (ROS) (11, 12). Elevated glucose levels hinder the transition from pro-inflammatory M1 macrophages to anti-inflammatory M2 macrophages, thereby disrupting the resolution of inflammation. Additionally, high glucose concentrations provide increased nutrient availability, promoting bacterial growth and proliferation, which escalates the risk of infection. Furthermore, diabetic wounds often exhibit dysregulated cytokine release by neutrophils during the inflammatory phase, thereby creating a favorable environment for wound infection (13). Beyond this elevated glucose levels rigidify cell membranes and constrict blood vessels, thereby reducing blood flow and decreasing the availability of nutrients and oxygen at the wound site (14). Elevated glucose levels promote the overexpression of matrix metalloproteinases (MMPs) such as MMP-9 and MMP-2, leading to excessive ECM degradation, impaired proliferation of keratinocytes and fibroblasts, and hindered angiogenesis. However, certain MMPs, like MMP-8, can be beneficial for diabetic wound healing (15). Elevated blood glucose levels, a key characteristic of diabetic wound environments, represent the primary barrier to effective healing. Thus, the primary focus in the clinical treatment of diabetic wounds is to manage blood sugar levels effectively (14).

Hypoxia in diabetic patients results from restricted oxygen supply and increased oxygen consumption in the wound. Vascular dysfunction and neuropathy limit oxygen delivery to the wound site. Reduced blood oxygen levels lead to decreased expression of hypoxia-inducible factor-1α (HIF-1α) and its target genes, impairing the cellular response to hypoxia. This disruption affects angiogenesis, resulting in delayed wound healing (16). Hypoxic environments negatively affect various cells, including ECs, macrophages, keratinocytes, and fibroblasts. Consequently, hyperbaric oxygen therapy has been extensively used in the treatment of diabetic foot ulcers (DFUs), although the exact mechanisms of hyperbaric oxygen treatment remain unclear (17).

Oxidative stress arises from an overproduction of ROS and insufficient antioxidant defenses. Hyperglycemia is a key mechanism for inducing oxidative stress, and ROS are crucial regulators of several phases of wound healing (12). Low levels of ROS are necessary to defend against external damage (18). However, excessive oxidative stress and reduced antioxidant capacity are major contributors to non-healing diabetic wounds (19). These factors disrupt normal healing stages by causing abnormalities and as a result, wounds can experience impaired healing progression, leading to numerous clinical complications (6, 20, 21). Importantly, diabetic ulcers stand as the primary reason for lower limb amputations (22). Currently, 10.5% of the global adult population between the ages of 20 and 79 have diabetes, amounting to 537 million people worldwide. Estimates suggest this figure will escalate to 643 million by the year 2030 and further increase to 783 million individuals living with diabetes by 2045 (23, 24). Current basic treatments for diabetic wound healing include blood sugar control, debridement, and infection control using topical or systemic antibiotics. Advanced therapies also play a significant role, including the development of skin substitutes, negative pressure wound therapy, and hyperbaric oxygen therapy. Additionally, new wound dressings that incorporate growth factors and bioengineered tissues are being utilized to enhance the healing process (25, 26).

Despite the availability of treatments for wound healing in diabetic patients, the persistently elevated rate of negative outcomes underscores the critical necessity for pioneering therapeutic methods. In this review, we focused on the role of non-coding RNAs (ncRNAs), including microRNAs (miRNAs), long non-coding RNAs (lncRNAs), and circular RNAs (circRNAs) in diabetic wound healing explaining their roles in this specific process to enhance our comprehension of the complex mechanisms behind DFUs and to devise novel approaches to facilitate the restoration of wounds in diabetic individuals.

## 2 Non-coding RNAs

NcRNAs, making up 99% of the RNA content in cells, play a pivotal role in influencing specific cellular responses. NcRNAs achieve this by modifying the expression or function of a wide array of downstream targets (27). Depending on the size of their nucleotide sequences, ncRNAs are categorized into two main groups: short ncRNAs comprising less than 200 nucleotide units, and lncRNAs, which are composed of nucleotide chains exceeding 200 units in length (28). NcRNAs operate via diverse molecular processes, such as regulating the expression of specific genes, affecting protein function and activity, and interacting with relevant signaling pathways. Any abnormalities in their expression can lead to the onset of numerous diseases (29, 30). NcRNAs demonstrate connections to numerous disorders linked to diabetes mellitus (DM), including DFUs, diabetic nephropathy, diabetic cardiomyopathy, and diabetic peripheral neuropathy, through their interactions with proteins. Despite this, the specifics of how ncRNAs operate and exert their effects remain largely unknown, leaving many questions about their mechanisms of action unanswered (31–33). While all three types of ncRNAs have both beneficial and detrimental effects at different stages of diabetic wound healing, there are some major differences among them. LncRNAs, in contrast to miRNAs, are longer (over 200 nucleotides in length) and regulate gene expression at various levels, including chromatin modification, transcription, and post-transcriptional processing. They can also interact with and act as sponges for miRNAs to regulate gene expression (34). CircRNAs are unique among these three types due to their closed-loop structure, which gives them resistance to degradation, making them more stable and potentially better biomarkers for different diseases (35). Beyond their role in diabetic wound healing, ncRNAs serve as important resources for diagnosing and potentially treating a range of diseases. These include various types of cancer, cardiovascular diseases, rheumatoid arthritis, tuberculosis, kidney stones, and eye disorders (36–41).

## 3 Mi-RNAs

MiRNAs belong to a class of endogenous, small molecules of ncRNAs, which serve as the main modulators in the post-transcriptional regulation of gene expression. These molecules carry out their regulatory roles by specifically interacting with the three prime untranslated regions (3’-UTR) of target messenger RNAs (mRNAs) (42, 43), leading to mRNA cleavage, translational repression, or mRNA deadenylation. This intricate mechanism allows miRNAs to control a wide array of biological processes, making them crucial components in the cellular regulatory networks (44–47). The examination of miRNAs expression levels can significantly contribute to the diagnosis of various diseases (48, 49). For instance, the expression of miR-129 and miR-335 is notably reduced in the skin tissues of patients suffering from DM (50). Similarly, miR-296-5p expression is substantially reduced in DM tissues, compared to normal tissues (51). Furthermore, miR-488-3p levels are reduced within the wound tissues of individuals afflicted with DM (52). Conversely, upregulation of miR-222-3p has been obtained from individuals diagnosed with Type 2 diabetes mellitus (T2DM), and a decrease of miR-126-3p within circulating plasma has been documented among individuals suffering from diabetes mellitus (53, 54). In addition, miRNAs play a significant role in modulating the functionality of β-cells, adjusting the expression of genes crucial for maintaining pancreatic β-cell homeostasis (55, 56).

### 3.1 Inflammation

At the inflammatory stage, macrophages are the key contributors and exhibit significant flexibility, being able to differentiate into diverse phenotypic cells based on the environmental shifts they encounter (57). In diabetic wound healing the process is often impeded at the inflammatory phase due to the impaired shift from M1 to M2 macrophage (58, 59). Pro-inflammatory M1 macrophages are predominant in diabetic wound environments, with minimal presence of anti-inflammatory M2 macrophages. M1 macrophages release pro-inflammatory cytokines, including IL-1β, IL-6, IL-12, IL-18, IL-23, and TNF, which impede diabetic wound healing. In contrast, M2 macrophages produce anti-inflammatory cytokines such as IL-10 and IL-12, as well as growth factors like TGF-β, which facilitate wound healing. Therefore, maintaining a balance between M1 and M2 macrophages is crucial in the diabetic wound healing process (13). Unlike macrophages, neutrophils that evolve into neutrophil extracellular traps (NETs) exacerbate the wound’s inflammatory reaction, adversely affecting diabetic wound healing (60). Here we explore the molecular processes through which various types of miRNAs exert regulatory control over the inflammatory phase.

Multiple research endeavors have reported a reduction in miR-146a levels within macrophages of diabetic patients, underscoring the pivotal function miR-146a plays in regulating the inflammatory stage of impaired wound repair processes (61, 62). This functional significance is noted in the regulation of nuclear factor-kappa B (NF-κB) activation and the interleukin-1 receptor-associated kinase 1 (IRAK1) pathway (63, 64). Investigations into the therapeutic potential of conjugating cerium oxide nanoparticles (CNP) to miR-146a have demonstrated significant benefits in diabetic wound repair (61, 65). Li et al. explored a novel dressing approach by encapsulating miR-146a within exosomes and incorporating these exosomes into a silk fibroin patch (SFP). This innovative dressing demonstrated efficacy in downregulating the expression of IRAK1, Tumor Necrosis Factor-Alpha (TNF-α), Interleukin-1 beta (IL-1β), and Interleukin-6 (IL-6) *in vitro* in HaCaT cells, as well as IRAK1, and phosphorylated NFκB-p65 in diabetic mice, thereby promoting wound healing (66). Zhou et al. discovered that EXO-miR-146a shifts of macrophages toward the anti-inflammatory M2 macrophage by reducing TNF Receptor Associated Factor 6 (TRAF6) expression under high glucose conditions (67). In summary, miR-146a promotes diabetic wound healing by targeting and regulating key inflammatory molecules and pathways.

Research findings have indicated that miR-145a-5p exerts a beneficial influence on the wound repair processes in diabetic conditions promoting the polarization towards M2 macrophage (68, 69), through modulating the activity of the p21-Activated Kinase 7 (PAK7) and influencing β-catenin signaling in hyperlipidemia (69). The research conducted by Su and colleagues revealed that miR-145-5p, transferred through extracellular vesicles (EVs), directly modulates the expression of the cyclin-dependent kinase inhibitor 1A (CDKN1A), consequently activating the Erk/Akt, thus promoting wound healing in high glucose (HG)-induced human dermal fibroblasts as well as in murine models of diabetes mellitus *in vivo* (70). Contrarily, Wang and colleagues demonstrated that miR-145-5p exerts a detrimental influence on HG-induced impairment in human foreskin fibroblasts and hinders wound repair proven in murine models of diabetes mellitus. Furthermore, they showed that suppressing the expression of miR-145-5p facilitates an improvement in cellular functionality within fibroblast cultures and promotes the wound repair processes in DFUs mouse models through upregulating platelet-derived growth factor D (PDGFD) expression (71). These studies suggest that miR-145a-5p exhibits both beneficial and detrimental effects on diabetic wound healing.

Ban et al. revealed that treatment with miR-497 leads to a decrease in IL-1β, IL-6, and TNF-α levels thereby facilitating the wound healing process (72). MiR-21 was found to have an inhibition effect on the inflammatory response triggered by lipopolysaccharide (LPS) and increases IL-10 production by interacting with phosphatase and tensin homolog (PTEN) and programmed cell death 4 (PDCD4) genes (73). In other words, these miRNAs promote diabetic wound healing by regulating pro-inflammatory and anti-inflammatory cytokines.

NF-κB serves as a crucial regulator of the inflammatory response by controlling the expression of genes related to pro-inflammatory proteins such as cytokines, chemokines MMPs, growth factors, and proteins involved in apoptosis. Dysregulation of NF-κB leads to the development of inflammatory diseases (74). Consequently, targeting the NF-κB pathway has been proposed as a promising therapeutic strategy for managing diabetic complications. miR-185-5p and miR-132 have been found to have beneficial effects in diabetic wound healing by targeting NF-κB. Wang et al. observed a downregulation of miR-185-5p in the wound tissues of both DFU patients and diabetic rats, with the administration of miR-185-5p mimics shown to accelerate wound repair by inhibiting the expression of NF-κB and intercellular adhesion molecule 1 (ICAM-1) in diabetic rat wound tissue (75). Furthermore, The study by Ge et al. suggests that miR-132-exo promotes diabetic wound healing and improves the viability of skin flaps by facilitating M2 macrophage polarization and enhancing angiogenesis. The M2 macrophage polarization induced by miR-132-exo may be mediated by inhibiting the NF-κB signaling pathway through the suppression of p65 (76). Yang et al. revealed that miR-203a-3p targets inhibitor of cytokine signaling 3 (SOCS3), thereby activating the JAK2/STAT3. This activation leads to M2 macrophage polarization and subsequently enhances diabetic wound repair (77).

### 3.2 Angiogenesis

Angiogenesis, which involves generating novel blood vessels from existing ones, is a process that demands a carefully controlled mix of signals that either stimulate or inhibit (78–80). Diabetic wounds often have an impaired blood supply due to insufficient angiogenesis (81). Several proangiogenic factors, notably VEGF, FGF, and Epidermal Growth Factor (EGF), serve vital functions in angiogenesis (82, 83). VEGF plays a crucial role in regulating angiogenesis and vascular permeability. In diabetic patients, VEGF levels in the serum and vitreous are associated with blood glucose control (84).

Vascular Endothelial Growth Factor A (VEGFA) is the most studied member of the VEGF family. Research revealed an elevated presence of miR-195-5p and miR-205-5p in EVs from DFU wound fluid, and these miRNAs inhibit angiogenesis by directly targeting the 3′-UTR region of VEGFA mRNA, thereby regulating VEGFA expression (85). MiR-135a-3p negatively regulates angiogenesis, controlling the VEGF-induced activation of the p38MAPK pathway by targeting huntingtin interacting protein 1(HIP1) (86). MiR-615-5p was identified as a key inhibitor of angiogenesis in ECs by impacting the VEGF-AKT/eNOS and specifically interacting with the insulin-like growth factor 2 (IGF2) and Ras association domain-containing protein 2 (RASSF2) genes (87). Wang et al. detected upregulated levels of miR-199a-5p in the wound tissues of patients with DFUs. Subsequently, they demonstrated that in diabetic rats, the suppression of miR-199a-5p enhanced the VEGFA expression and Rho-associated protein kinase 1 (ROCK1), thereby promoting wound healing (88). In contrast, Zhou et al. demonstrated that the inhibition of hsa-miR-199a-5p by SNHG12/NFYC-AS1 promotes diabetic wound healing via the hsa-miR-199a-5p-S100A8/S100A7/XDH pathway (89).

Under diabetic conditions, HIF-1 upregulates the expression of VEGF highlighting the critical role of HIF-1 regulation in angiogenesis (90). Xiao et al. demonstrated that overexpression of miR-1248 promotes wound healing by targeting Cbp/p300-interacting transactivator 2 (CITED2) and subsequently modulating the activity of HIF-1 (91). HIF-1 has been also found to be associated with miR-31-5p in its mechanism of promoting angiogenesis in diabetic wound healing. Several studies have underscored the significant contribution of miR-31-5p in promoting angiogenesis by targeting factor-inhibiting HIF-1 (HIF1AN) (92, 93). Furthermore, it was successfully demonstrated that miR-31-5p encapsulated in exosomes promotes diabetic wound healing by enhancing angiogenesis (94). In patients with DFU, the expression of miR-15a-3p was found to be upregulated in foot skin compared to control subjects without the condition. miR-15a-3p appears to modulate the NOX5/ROS signaling pathway, suggesting a specific mechanistic pathway through which miR-15a-3p impacts diabetic wound healing (95).

There are multiple studies introducing the beneficial role of miR-221-3p in different phases of diabetic wound healing including angiogenesis. MiR-221-3p promotes angiogenesis by directly targeting homeodomain-interacting protein kinase 2 (HIPK2), utilizing both *in vitro* experiments with HUVECs and in diabetic mice skin tissues (96). Similarly, Hu et al. demonstrated that miR-221-3p enhances skin wound healing by mitigating the negative effects of HG on apoptosis and angiogenesis, specifically through targeting thrombospondin 1 (THBS1) (97). MiR-200b was observed to be upregulated in HUVECs treated with HG, and miR-200 inhibition was found to promote angiogenesis in HUVECs through the interaction with the neurogenic locus notch homolog protein 1 (Notch1) (98). The Wnt/β-catenin signaling pathway is critical for diabetic wound healing, regulating essential processes such as cell proliferation, migration, and differentiation of keratinocytes and fibroblasts, while also promoting the activity of ECs. However, in diabetic wounds, the significant decrease in β-catenin pathway activity leads to impaired wound closure and chronicity, largely due to reduced cytokine production and dysfunctional intracellular signaling that hinders the functionality of key cell types (57, 99). MiR-488-3p accelerates wound healing by activating the cytochrome P450 1B1 (CYP1B1)-mediated Wnt4/β-catenin through targeting MeCP2, thereby promoting the angiogenic response of HUVECs (52).

Blood glucose levels regulate the expression of PTEN, predominantly present in epithelial cells, and it triggers signaling pathways influencing angiogenesis (46). Xu et al. demonstrated that the levels of PTEN are downregulated in individuals with diabetes, and that suppression of miR-152-3p can elevate PTEN levels, thereby improving ECs functions critical to angiogenesis (100). Moreover, sEVs engineered with miR-17-5p, when encapsulated in GelMA Hydrogel, promote diabetic wound healing by stimulating angiogenesis and collagen buildup through PTEN and p21 pathways (101). MiR-17-5p belongs to the miR-17-92 cluster, which regulates ECs functions and modulates angiogenesis through various pathways. However, the roles of the miR-17-92 cluster are not always beneficial (102, 103). Another member of the miR-17-92 cluster, miR-17-3p, has been found to negatively control fetal liver kinase 1 (Flk-1) expression. The miR-17-3p/Flk-1 interaction has a detrimental effect on angiogenesis under diabetic conditions (104). Similarly, miR-92a, another member of the miR-17-92 cluster, has been associated with detrimental effects on angiogenesis. Inhibition of miR-92a has been found to have a beneficial effect on angiogenesis, thereby improving diabetic wound healing. Lucas et al. demonstrated that light-activated antimir-92a improves angiogenesis under diabetic conditions (105, 106). Furthermore, another study demonstrated that a synthetic miR-92a inhibitor (MRG-110) promotes angiogenesis and granulation tissue formation, thereby accelerating wound healing in both mice and pigs (106). These results suggest that the regulatory role of the miR-17-92 cluster is complex and varied, necessitating further research to fully understand their mechanisms in angiogenesis and harness their potential for therapeutic purposes.

The research by Wang and colleagues showed that miR-503 from M1 macrophage-released EVs inhibits insulin-like growth factor 1 receptor (IGF1R) expression in HUVECs, thereby impeding the wound healing process in individuals with diabetes (107). Elevated levels of miR-409-3p were noted in nonhealing skin wound samples from patients relative to uninjured skin tissues. Additionally, miR-409-3p was shown to impair the angiogenic capabilities of HUVECs under high glucose conditions, indicating that miR-409-3p exerts a detrimental effect on wound healing in hyperglycemic environments by altering the BTG2/mTOR signaling pathway (108).

### 3.3 Re-epithelialization and ECM remodeling

Re-epithelialization and ECM remodeling are key processes in the regenerative phase. Re-epithelialization, essential for restoring intact skin, is primarily facilitated by keratinocytes through their migration, proliferation and differentiation, thereby maintaining and repairing skin function (109). The formation of the ECM holds significant importance in diabetic wound healing (58). In healthy skin, fibroblasts migrate to the injury site to restructure the ECM and remodel dermal collagen. However, in diabetic skin, under glycemic conditions, these cells exhibit decreased proliferation and migration, leading to the impairment and fragmentation of collagen fibers (110).

MiR-155 has been found to be overexpressed in HaCaT cells under high glucose (HG) conditions and in the exosomes of M1 macrophages. Gondaliya et al. demonstrated that MSC-derived exosomes loaded with a miR-155 inhibitor promote wound healing *in vitro* with HaCaT cells and *in vivo* in diabetic mice, primarily by restoring FGF-7 levels, thus enhancing keratinocyte migration and re-epithelialization (111). FGF, including FGF-7, play a crucial role in accelerating the healing process of diabetic wounds. However, despite their potential, the clinical application of FGF in treating diabetic wounds is limited, highlighting the need for improved delivery methods and further research into their long-term effects (112). Relatedly, Moura et al. demonstrated that in hyperglycemic conditions, an miR-155 inhibitor promotes wound closure and re-epithelialization *in vitro* by facilitating scratch closure and upregulating FGF7 expression (113). In their experiments, Dallas et al. utilized an antisense oligonucleotide (antimiR) to inhibit miR-210, mitigating its negative impact on keratinocyte growth and proliferation and thus promoting re-epithelialization (114).

Multiple studies have identified the role of miR-145-5p in various stages of diabetic wound healing. Wang et al. showed that miR-145-5p adversely impacts HG-induced impairment in human foreskin fibroblasts by elevating PDGFD expression, consequently hindering wound healing (71). Conversely, Su et al. found that EVs from placental mesenchymal stem cells counter apoptosis and enhance the proliferation and migration in diabetic wounds by delivering miR-145-5p. MiR-145-5p targets CDKN1A directly, consequently upregulating the Erk/Akt cascade, and thus promoting diabetic wound healing (70). MiR-4645-5p, derived from human bone marrow stem cells (hyBMSCs), was discovered to promote autophagy in HaCaT cells by suppressing the MAPKAPK2-induced AKT-mTORC1, consequently aiding diabetic wound healing (115).

MMPs play a crucial role in the regulation of keratinocytes, and thereby in re-epithelialization and ECM remodeling (116). Li et al. discovered that inhibiting PPARG, targeted by miR-182-5p, results in reduced MMP1 expression and elevated fibronectin 1 (FN1) expression. This suggests that exosomes from human endothelial progenitor cells improve the proliferation, migration, and adhesion of HaCaT cells in HG conditions via the miR-182-5p/PPARG signaling pathway (117). Furthermore, another study by Wang and colleagues found that the upregulation of miR-129 and miR-335 inhibits MMP-9 expression by interacting with specificity protein 1 (Sp1) in keratinocytes and in diabetic rats, thereby promoting diabetic wound healing (50).

**Figure 2:**
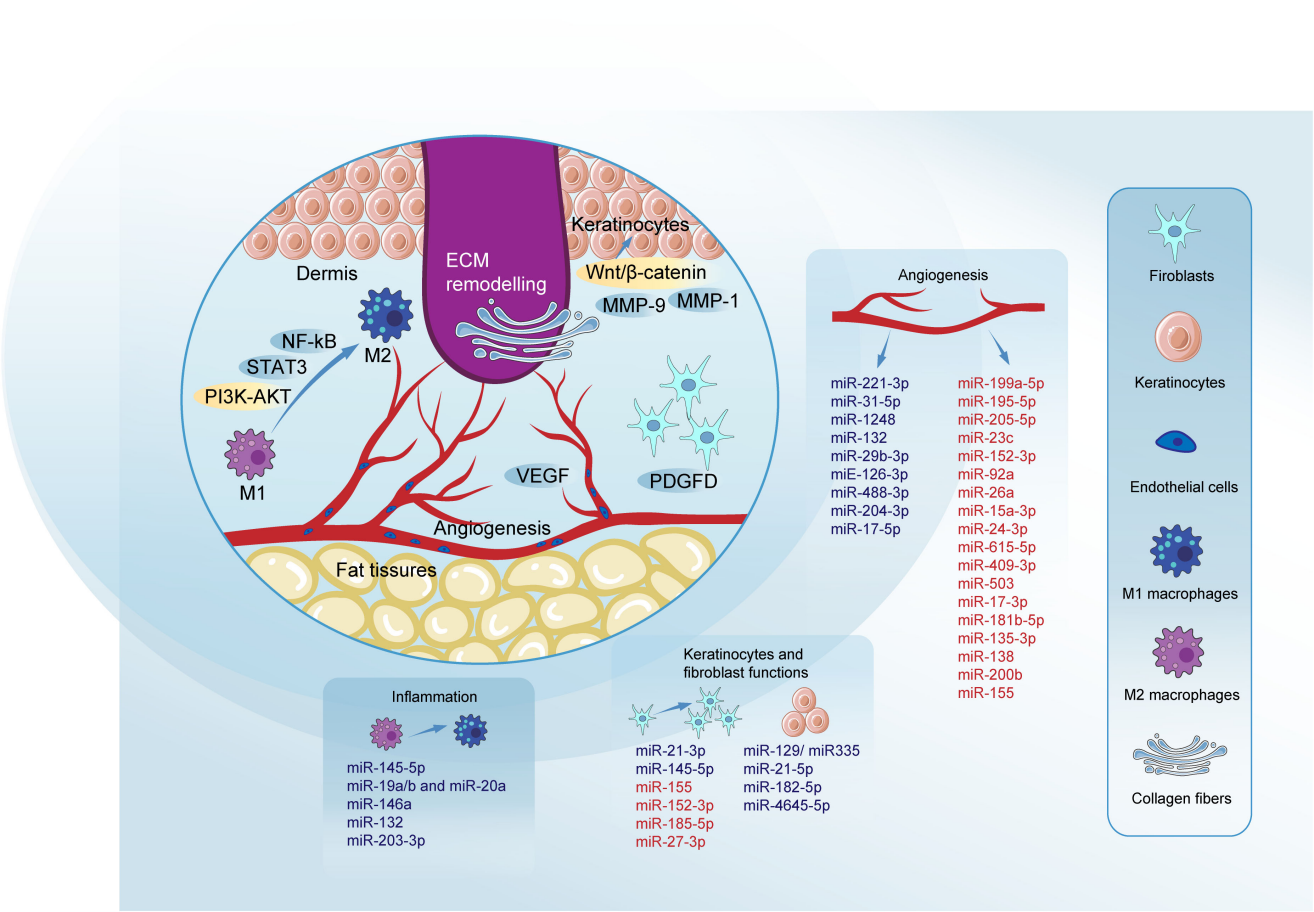
Roles of miRNAs in key phases of diabetic wound healing. MiRNAs modulate key pathways to promote wound healing. The inhibition of NF-κB and activation of the PI3K/AKT signaling pathway are critical, with signal transducer and activator of STAT3 playing a pivotal role in promoting M2 macrophage polarization through pathways such as DYRK1A/STAT3 and JAK2/STAT3. VEGF regulation is crucial, involving pathways like HIF1A/VEGF, VEGF-AKT/eNOS, and VEGF-induced activation of the p38MAPK signaling pathway, which facilitates blood vessel formation. The Wnt/β-catenin pathway is essential for the proliferation and migration of keratinocytes and fibroblasts, crucial for effective re-epithelialization of the wound. MMPs such as MMP-9 and MMP-1 play dual roles in diabetic wound healing. Their effects can be beneficial or detrimental depending on the healing phase. PDGFD is highlighted for its positive impact on fibroblast proliferation and migration, contributing to effective ECM remodeling. Blue denotes miRNAs promoting specific processes, while red indicates those impairing them. Micro RNAs (miRNAs); signal transducer and activator of transcription 3 (STAT3); phosphatidylinositol-3 kinase-AKT (PI3K-AKT); signal transducer and activator of transcription 3 (STAT3); nuclear factor-kappa B (NF-kB); matrix metalloproteinase-1 (MMP-1); matrix metalloproteinase-9 (MMP-9); platelet-derived growth factor D (PDGFD); extracellular matrix (ECM); vascular endothelial growth factor (VEGF); epidermal growth factor (EGF); fibroblast growth factor (FGF).

Different members of the miR-21 family have been found to positively affect the functions of keratinocytes and fibroblasts, thereby promoting diabetic wound healing. MiR-21-5p loaded in engineered exosomes enhances the proliferation and migration of HaCaT cells via the Wnt/β-catenin pathway *in vitro* and enhanced diabetic wound healing by promoting collagen remodeling, and vascularization in diabetic rats (118). Furthermore miR-21-5p was found to be involved in wound repair through Centella Asiatica and its compound Asiaticoside. The study by Liu et al. concludes that Asiaticoside-nitric enhances diabetic wound healing by modulating the miRNA-21-5p/TGF-β1/SMAD7/TIMP3 cascade (119). Wu et al. found that miR-21-3p plays a positive role in fibroblast function by downregulating sprouty homolog 1 (SPRY1) and accelerates wound healing (120). Neuro-oncological ventral antigen 1 (NOVA1) has been identified as a direct target of miR-27-3p in fibroblasts, and inhibiting miR-27-3p could promote diabetic wound healing by restoring fibroblast viability (121). We summarized the regulatory mechanism of several key miRNAs in diabetic wound healing (**Table 1**, **Figure 2**).

**Table 1:** Role of miRNAs in the regulation of diabetic wound healing.

miRNAs	Expressions	Animal models	Regulation mechanisms	References
miR-31-5p	Significant downregulation of miR-31-5p compared with nondiabetic wounds	1) Mice were intraperitoneally injected with STZ (50 mg/kg) for 5 days. Full-thickness excision wound at a diameter of 8 mm was performed on the back of all mice 2) Rats were injected STZ dissolved in 0.1 M phosphate-citrate buffer a dose of 55 mg/kg. Full-thickness skin wounds were generated (diameter = 2.0 cm)	Promotes Angiogenesis proliferation and re-epithelialization by inhibiting HIF-1	1) (92) 2) (94)
miR-19a/b and miR-20a	Lower in chronic ulcers than in acute wounds at the proliferative phase	Mice received multiple injections of low-dose STZ to induce diabetes.	Combinatory treatment with miR-19b and miR-20a decrease TLR3-mediated NF-dB activation by targeting SHCBP1 and SEMA7A, respectively, reducing the inflammation	(103)
miR-199a-5p	Significantly Increased in wound tissues of patients with diabetic foot ulcers compared with nondiabetic wounds	Rats received injection with a dose of 5 mL/kg through the abdominal cavity. piece of 2 cm round whole-layer skin was removed from both sides of the back	Inhibition of miR-199a-5p rescued impaired proliferation and migration of HG induces cells by enhancing the expression of VEGFA and ROCK1	(88)
miR-129-2-3p	MiR-129-2-3p in diabetic-derived neutrophils was downregulated to less than one-third of the level in nondiabetic-derived neutrophils	Male mice aged 8 to 12 weeks were used. Full-thickness excisional dorsal wounds (4 mm) were created using a biopsy punch.	Influences the behavior of neutrophils, affecting inflammatory responses and apoptosis by regulating Casp6, Ccr2, and Dedd2	(197)
miR-1248	Decreased in the hADSCs of DM patients	Rats were administered a single intraperitoneal injection of 60 mg/kg body weight of STZ dissolved in citrate buffer (0.1 mol/L, pH 4.5). A wound was created with a 1-cm circular diameter and a depth reaching the hypodermis.	Promotes angiogenesis and proliferation by targeting CITED2 and regulating HIF-1α expression	(91)
miR-23c	1.5-fold increase in patients with infected DFU	N/A	Inhibits Angiogenesis by targeting SDF-1α	(198)
miR-152-3p	Overexpressed in DFU patients	1. A high-fat diet combined with intraperitoneal injection of STZ (50 mg/kg) for 5 consecutive days. Rectangular wounds (2 mm × 5 mm) were made on the right instep surface 2. Full-thickness cutaneous skin wound (10 mm in diameter) was produced on male diabetic mice (db/db) mice	1. MiR-152-3p suppression inhibited the apoptosis of fibroblasts and promoted their migration by activating the FBN1/TGF-β 2. Impedes angiogenesis by Increasing PTEN levels	1) (199) 2) (100)
miR-26a	Increased levels in ECs treated with D-glucose	Db/db mice with a full-thickness skin wound (1cm2)	Inhibition of miR-26a promotes angiogenesis through BMP/SMAD1-ID1 signaling pathway	(200)
miRNA-497	Significantly downregulated in diabetic wounds on mice compared to the normal wounds	Diabetes was induced by intraperitoneal injection of STZ (100mg/kg) in 0.01 M citrate buffer (pH 4.5) twice a week during the fasting state. Full-thickness excisional wounds were created on the dorsal skin of mice with an 8-mm biopsy punch	Decreases the levels of pro-inflammatory cytokines by reducing the levels of IL-1β, IL-6, and TNF-α	(72)
miR-185-5p	Downregulated in wound tissues of diabetic foot patients	After two weeks of adaptive feeding, the rats were accepted 40 mg/kg STZ by intraperitoneal injection. A 1.0 cm diameter full-thickness circular wound was made on the dorsal skin	Inhibits prolonged inflammation by suppressing NF-κB and ICAM-1	(75)
miR-146a	Decrease in miR-146a expression in the macrophages of patients with diabetes	1) Db/db mice with full-thickness wounds on the back. 2) Mice were intraperitoneally injected with 1% STZ. A deep incision was made in the front midline of the back of the mice	1) Promotes anti-inflammatory response by targeting IRAK1 and inhibiting NF-κB 2) Promotes M2 macrophage polarization by inhibiting the TLR4/NF-κB	1) (66) 2) (62)
miR-129 and miR-335	Both are downregulated in diabetic skin tissues	Diabetes was induced through 50 mg/kg of STZ intra-abdominal injection after 12 h without provision. Then, full-thickness excision wounds were created on the middle of the back of each Rat	Promotes keratinocyte proliferation, migration and recovered collagen content by Inhibiting Sp1-Mediated MMP-9 Expression	(50)
miR-21-3p	Decreased in patients with diabetes as compared with those in the healthy control	Full-thickness cutaneous skin wounds (10 mm diameter) were created on male db/db mice	Promotes fibroblast proliferation and migration by downregulating SPRY1	(120)
miR-221-3p	Overexpression at the wound edge of normal mice	1) A diabetic mouse model was created by administering STZ intraperitoneally at a dose of 50 μg/g per day for 5 consecutive days. Full-thickness skin wounds with a diameter of 6 mm were then created.2) Mice received an injection of STZ (50mg/kg) for 5 consecutive days. Two full-thickness dermectomy wounds were symmetrically generated near the dorsal midline using a 6mm biopsy punch 3) Mice were injected intraperitoneally with STZ (50 mg/kg body weight) daily for 5 days. 6 mm biopsy punch was used to produce two full-thickness dermal excisional wounds	1) Targets HIPK2 and promotes viability, migration, and tube formation of HUVECs 2) Inhibits apoptosis in HaCaT cells, and enhanced angiogenesis in HUVECs by targeting THBS1 3) Inhibits the inflammatory response of keratinocytes by modulating DYRK1A/STAT3 signaling pathway	1) (96) 2) (97) 3) (201)
miR-145-5p	Downregulated during the tissue formation stage. Decrease in hyperlipidemia rat models.	1) Rats were daily fed a normal or 2% cholesterol along with 0.25% cholate-enriched rat chow for a continuous 12 weeks 2) Db/db mice with full-thickness excisional skin wounds on the dorsum (10 mm in diameter)	1) Promotes M2 macrophage polarization by targeting PAK7 and regulating β-catenin signaling 2) Inhibits apoptosis and induces proliferation and migration of fibroblasts by targeting CDKN1A and activating the Erk/Akt signaling pathway.	1) (69) 2) (70)
miR-195-5p and miR-205-5p	Upregulated in in DF-EVs	Male rats with a wound in the dorsal skin	Inhibits angiogenesis by suppressing the expression of VEGFA	(85)
miR-15a-3p	MiR-15a-3p levels in foot skin were higher in DFU patients compared to the control group	Male six-week-old mice weighing 20-30g. Full-thickness excisional skin wound (10 mm in diameter) was produced on the upper back of each mouse	Impairs HUVEC functionality by targeting the NOX5/ROS signaling pathway	(95)
miR-27-3p	Overexpressed in cutaneous fibroblasts of diabetic patients	6–8-week old mice with full-thickness dorsal wounds (10 x 10 mm2)	Suppresses proliferation and migration of fibroblasts by targeting NOVA1	(121)
miR-24-3p	Upregulated in diabetic exosomes	The upper back of mice (weighing 25‐35 g and 8 weeks old) was given one full‐thickness skin excision with a diameter of 10 mm	Inhibits HUVEC proliferation, migration, and angiogenesis by targeting PIK3R3	(202)
miR-155	Enhanced miR-155 levels in HaCaT cells under HG conditions as compared to normal glucose conditions. Highly expressed in M1 exosomes	1) Male mice were administered a low dose of STZ, 50 mg/kg, for 5 consecutive days. The wound was created using a 4 mm biopsy punch 2) Two 6 mm excisional wounds 2 cm apart were created on mice by using a punch biopsy tool 3) Two full-thickness wounds extending through the panniculus carnosus were created on the dorsum on each side of midline of db/db mice	1) MiR-155-inhibitor-loaded MSC-derived exosomes promote re-epithelialization and angiogenesis by modulating the expression levels of FGF-7, VEGF, and matrix metalloproteinases 2) MiR-155 inhibitor enhanced scratch closure in hyperglycemic conditions by increasing FGF7 expression, promoting re-epithelialization and wound closure 3) Suppresses endothelial cell functions and angiogenesis by targeting growth differentiation factor 6 (GDF6)	1) (111) 2) (113) 3) (203)
miR-615-5p	Increased in wounds of diabetic mice, in plasma of human subjects with acute coronary syndromes, and in plasma and skin of human subjects with diabetes mellitus	8–10 weeks old, db/db mice with full thickness skin wounds	Inhibits angiogenesis by targeting IGF2 and RASSF2 and suppressing VEGF-AKT/eNOS pathway	(87)
miR‐488‐3p	Lower in wound tissues of diabetics	Rats received an intraperitoneal injection of 50 mg/kg STZ. A full-thickness skin wound was created	Promotes proliferation and migration as well as angiogenic response of HUVECs through Wnt/β-catenin pathway by targeting MeCP2	(52)
miR-204-3p	Reduced expression in DFU patients and HG-treated HUVECs	N/A	Promotes HG-mediated proliferation, apoptosis, migration of HUVEC by targeting HIPK2	(204)
miR-409-3p	Increased in the nonhealing skin wounds of patients with type 2 diabetes compared to the non-wounded normal skin	The dorsal skin of the mice, along the median and 5–8 cm inferior to the skull, was abducted 1.25 cm using a pair of flat forceps and biopsied through to the dish with a 6-mm steel punch	Impairs the angiogenic capabilities of HUVECs by altering the BTG2/mTOR signaling pathway	(108)
miR-503	Upregulated in EVs derived from M1 polarized macrophages	Mice were given an injection of 50 mg/kg STZ. Two 8-mm full-thickness excisional wounds were made on the dorsum of each mouse	Inhibits the expression of IGF1R, leading to HUVEC dysfunctions	(107)
miR‐17‐3p	Increased in patients with DFUs who received maggot debridement therapy (MDT)	N/A	Inhibits tube formation through the downregulation of Flk‐1	(104)
miR-181b-5p	Upregulated in exosomes from DFU	Mice were fed a high-glucose and high-fat diet and intraperitoneally injected with STZ (45 mg/kg). Full-thickness excisional skin wound was inflicted on the upper backs of the mice	Promotes cell senescence and inhibits angiogenesis by regulating NRF2/HO-1	(205)
miR-138	Increased in DFU group rats compared with the control group	Diabetic rats were induced by single intraperitoneal injection of 60 mg/kg STZ dissolved in citrate buffer solution (0.1 M, pH 4.5). 4 × 4 mm wound depth reached fascia was made on the hind foot of the diabetic rats	Down-regulation of miR-138 alleviates inflammation by activating PI3K/AKT pathway and human telomerase reverse transcriptase (hTERT)	(86)
miR-200b	Upregulated in high glucose-treated HUVECs	N/A	Inhibition of miR-200b promotes angiogenesis by targeting Notch1	(98)

## 4 Long non-coding RNA

LncRNAs are sequences of RNA with a length exceeding 200 nucleotides and have many features in common with genes that encode mRNAs, yet they are incapable of synthesizing proteins (122, 123). LncRNAs exert a crucial regulatory influence on the processes of gene transcription and expression through a variety of molecular mechanisms, initially thought to act locally at their synthesis sites to influence nearby gene activity. These transcripts are involved in numerous mechanisms, such as protein synthesis, RNA maturation, stability, and transport, as well as activating or silencing transcriptional genes by modifying chromatin structure (124, 125). Furthermore, lncRNAs exert their influence on gene expression through mRNA splicing, translation, transcription, and genomic imprinting, showcasing their extensive regulatory capacity (125, 126). The link between lncRNAs and diseases, particularly chronic conditions like diabetes, cancer, and cardiovascular diseases, is clear and well-defined (127–130).

### 4.1 Inflammation

During the inflammatory stage, lncRNAs can serve a regulatory function by various pathways, including the M1 to M2 macrophage polarization (131). LncRNA H19 is the most extensively studied among all lncRNAs. It has been discovered by various studies that lncRNA H19 is significantly decreased in diabetic wounds compared to normal ones (132). Li and colleagues showed that exosomal lncRNA H19 targets miR-130b-3p, regulating PPARγ/STAT3 to promote M2 macrophage polarization. This process aids in promoting fibroblast proliferation and migration, and ECs angiogenesis, thus accelerating the wound healing process (133).

Hu et al. first detected that lncRNA GAS5 triggers the activation of the STAT1 in macrophages, and STAT1 is necessary for the expression of M1 marker genes. Furthermore, they demonstrated that the silencing of lncRNA GAS5 promotes diabetic wound healing. Taken together, their study suggests that a reduction in the levels of lncRNA GAS5 will promote impaired diabetic wound healing by facilitating M2 macrophage polarization (134).

Kuang et al. showed that miR-1914-3p targets milk fat globule-EGF factor 8 protein (MFGE8), influencing macrophage polarization through the TGFB1/SMAD3. Concurrently, miR-1914-3p is targeted by MALAT1, which binds miR-1914-3p competitively to suppress the TGFB1/SMAD3 pathway’s activity. Further studies revealed that KCs-Exo carrying MALAT1 modulates macrophage activities through the TGFB1/SMAD3 and enhances diabetic wound healing in mice (135). LncRNA Lethe has been identified to be involved in the control of ROS generation in macrophages by adjusting NADPH oxidase 2 (NOX2) gene expression through NF-κB signaling (136).

### 4.2 Angiogenesis

Multiple research findings have highlighted functional links between lncRNAs and the process of angiogenesis. Some nanomaterial delivery systems have played important advantages in drug delivery and therapy (137, 138). Tao et al. explored a new nano-drug delivery system targeting lncRNA using high-yield extracellular vesicle-mimetic nanovesicles (EMNVs), focusing on the effects of EMNVs enriched with lncRNA-H19 (H19EMNVs). Their findings revealed that H19EMNVs enhanced ECs functions, which were previously hindered by HG levels (132). LncRNA HOTAIR, carried by MSC-derived EVs, was observed to enhance angiogenesis in HUVECs *in vitro* through VEGF upregulation. Additionally, it facilitated the vascularization of the wound bed and improves wound healing in diabetic mice (139).

Under diabetic conditions HIF-1 enhances the expression of VEGF, indicating that the regulation of the HIF-1/VEGF pathway is crucial for diabetic wound healing. Additionally, multiple lncRNAs have been found to exert regulatory effects on this pathway (122). LncRNA GAS5 promotes diabetic wound healing by interacting with TATA-binding protein-associated factor 15 (TAF15) and triggering the HIF1A/VEGF in diabetic mice, while also enhancing the proliferation of HG-induced HUVECs *in vitro* (140). LncRNA ANRIL was found to be downregulated in DFU. Furthermore, ANRIL overexpression was shown to rescue the HG-impaired function of endothelial progenitor cells (EPCs) by regulating HIF1A mRNA stability through interaction with FUS. It was also discovered that alterations in ANRIL or HIF1A regulate VEGFA expression both in EPCs and in diabetic mice (141). The study by Han and colleagues revealed that lncRNA KLF3-AS1 enhances cell growth and tube formation, while simultaneously suppressing apoptosis in HUVECs under HG conditions, through the reduction in the expression levels of microRNA-383. Additionally, their research identified VEGFA as a direct target of miR-383. Moreover, KLF3-AS1 was found to significantly promote cutaneous wound healing in a diabetic mouse (142). Fu and colleagues explored how keratinocyte-derived exosomal lncRNA LINC01435 influences diabetic wound healing by affecting vascular ECs. Their research indicates that LINC01435 impedes the migration and tube formation capabilities of HUVECs, thereby restraining angiogenesis via the LINC01435/YY1/HDAC8 signaling pathway (143). Elevated levels of lncRNA DLEU1 were detected in the serum of patients with DFUs. Subsequent investigations revealed that miR-96-5p acted as a mediator in the detrimental effects of lncRNA DLEU1 on angiogenesis. Collectively, the findings indicated that lncRNA DLEU1 exerts its negative impact on HUVECs by suppressing cell proliferation, enhancing apoptosis, and increasing oxidative stress through its interaction with miR-96-5p (144).

### 4.3 Re-epithelialization and ECM formation

LncRNAs are involved in re-epithelialization and ECM remodeling, exerting regulatory control over the activities of keratinocytes and fibroblasts. Specifically, lncRNA-H19 has been demonstrated to enhance the proliferation and migration of HaCaT cells, which were pre-treated with 30 mM glucose for 24 hours, and to inhibit pyroptosis by inhibiting NOD-like receptor family pyrin domain-containing 3 (NLRP3) activation (145). Another study found that lncRNA-H19, particularly enriched in PDGFRα+ dermal fibroblasts, promotes fibroblast proliferation through the modulation of the p53-controlled cell cycle and modulates immune cell infiltration by reducing fibroblast-derived growth differentiation factor 15 (GDF15). Additionally, it was discovered that exosomes from Adipocyte Progenitor Cells (APCs) transport lncRNA-H19 to the injury sites in type 2 diabetes mice, thereby enhancing wound healing that was previously impaired (146). Li et al. found that lncRNA H19 recruits SRF to upregulate connective tissue growth factor (CTGF) levels, which enhances wound healing by promoting fibroblast proliferation and ECM remodeling, thereby accelerating wound healing in diabetic rats through the lncRNA H19/SRF/CTGF pathway (147). Silencing lncRNA H19 inhibits the functions of fibroblasts, with reduced fibrillin-1 (FBN1) but upregulates miR-29b. This led to poor expression of transforming growth factor-beta 1 (TGF-β1), Smad3, fibronectin (FN), and Col-1, and reduced ECM accumulation (148). Qian and colleagues showed that lncRNA-H19 sponges miR-19b by targeting SRY-related high-mobility-group box 9 (SOX9). This activation of SOX9 initiates the Wnt/β-catenin pathway, resulting in enhanced proliferation, migration, and invasion of human skin fibroblasts, thus facilitating wound healing (149). Guo et al. demonstrated that lncRNA H19 could recruit enhancer of zeste homolog 2 (EZH2) mediated histone methylation and regulate HIF-1α to enhance fibroblast activation, leading to the improvement of diabetic wound healing (150). Apart from the beneficial properties of lncRNA-H19, research conducted by Ji and colleagues discovered that reducing H19 levels could promote HaCaT cell proliferation and migration. This effect occurs as H19 competitively interacts with miR-17-5p, leading to an increase in RUNX1 expression (151).

It was detected that lncRNA CASC2 is downregulated in wound tissues of DFU patients. *In vitro*, lncRNA CASC2 facilitates wound healing by enhancing fibroblast migration, and *in vivo*, it accelerates wound healing in DFU mice. Furthermore, He et al. demonstrated that lncRNA CASC2 facilitates wound healing in DFU by promoting fibroblast migration and inhibiting apoptosis through the miR-155/HIF-1α pathway (152).

In recent studies, the functions of MALAT1 in regulating the epithelial-mesenchymal transition (EMT) of HaCaT cells have been elucidated. Zhang et al. demonstrated that the suppression of MALAT1 significantly decreases EMT induced by TGF-β1 (153). Additionally, they found that MALAT1 was involved in hyperglycemia-induced EMT in human HaCaT cells by interacting with miR-205 and promoting the levels of Zinc Finger E-box Binding Homeobox 1 (ZEB1) (154). The results indicate that MALAT1 could be a target for diseases characterized by aberrant EMT. Moreover, Shi et al. proposed using MALAT1 as a sponge for miR-142 to indirectly upregulate Sirt1 and nuclear factor erythroid 2-related factor 2 (Nrf2) for treating DFUs in elderly individuals (155). MALAT1 also enhances the activities of keratinocytes by functioning as a competing endogenous RNA (ceRNA), which involves competitive interactions with miR-106a-5p. Furthermore, zinc finger protein 148 (ZNF148)-activated MALAT1 upregulates ZNF148 levels through its competitive interaction with miR-106a-3p, creating a positive feedback loop that further boosts keratinocyte activity (156). Hong et al. demonstrated that the downregulation of lncRNA XIST levels suppresses the proliferation and migration of HG-induced HaCaT cells through the miR-126-3p/EGFR pathway (157).

**Figure 3:**
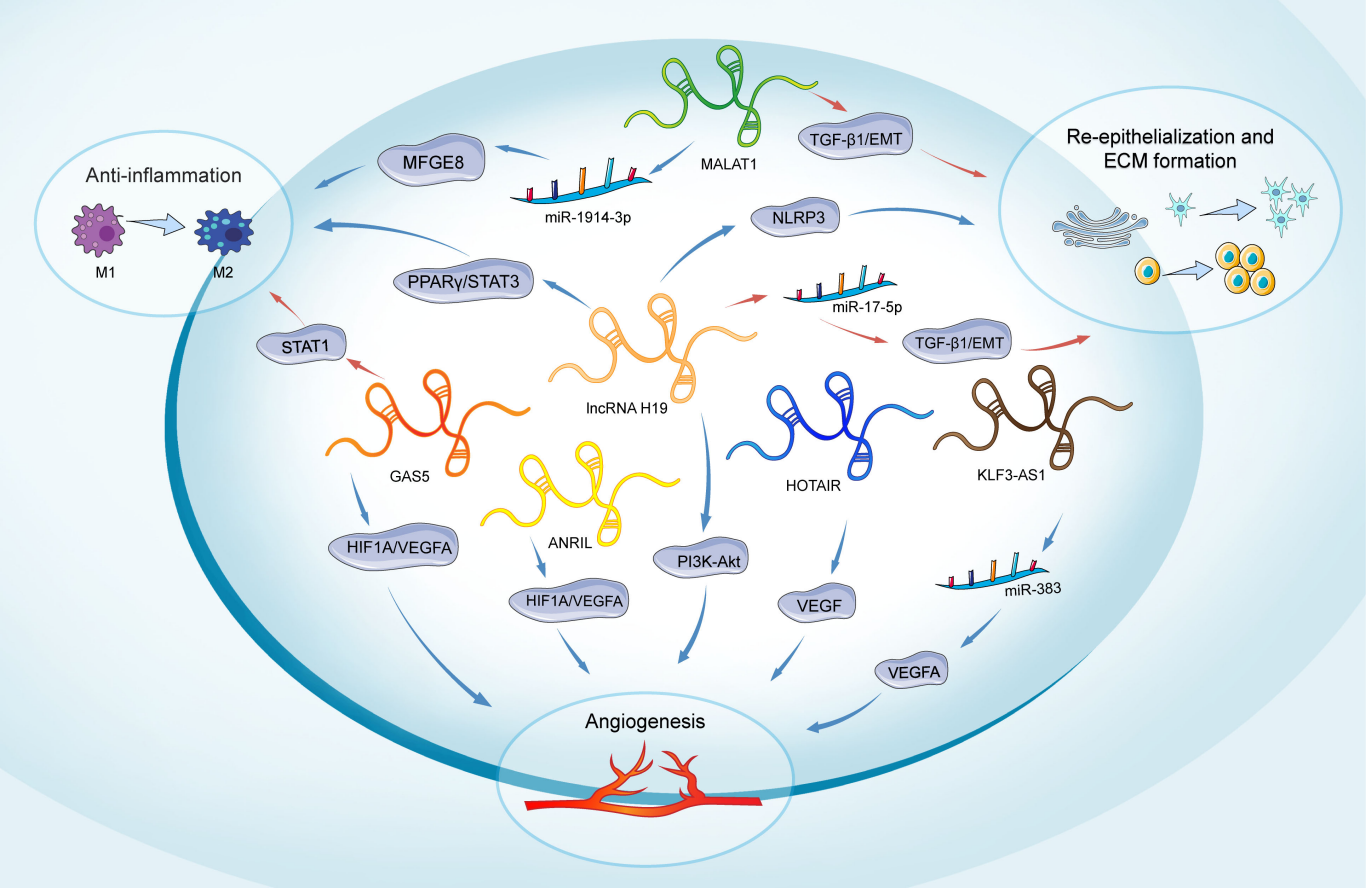
Role of lncRNAs in main phases of diabetic wound healing. The lncRNAs depicted include H19, MALAT1, GAS5, HOTAIR, ANRIL, and KLF3-AS1. H19 participates in all phases of wound healing. It promotes inflammation through the regulation of the PPARγ/STAT3 pathway. In angiogenesis, H19 regulates PI3K-Akt signaling. H19 enhances the proliferation and migration of keratinocytes and inhibits pyroptosis by suppressing the activation of NLRP3. Conversely, H19 can also inhibit keratinocyte proliferation and migration by targeting miR-17-5p and upregulating RUNX1. MALAT1 promotes an anti-inflammatory response by targeting miR-1914-3p and activating MFGE8. Additionally, MALAT1 regulates the EMT in keratinocytes through the regulation of TGFβ1-induced EMT. GAS5 induces STAT1 to promote M1 macrophage activation, while its reduction favors M2 macrophage polarization and benefits wound healing. It also promotes angiogenesis by activating the HIF1A/VEGFA pathway. HOTAIR, ANRIL, and KLF3-AS1 are primarily involved in promoting angiogenesis: HOTAIR activates VEGF to enhance angiogenesis, ANRIL modulates the HIF1A/VEGFA pathway, and KLF3-AS1 promotes angiogenesis by targeting miR-383 and upregulating VEGFA expression. Long non-coding RNAs (lncRNAs); extracellular matrix (ECM); milk fat globule-EGF factor 8 protein (MFGE8); peroxisome proliferator-activated receptor gamma/Signal transducer and activator of transcription 3 (PPARY/STAT3); Signal transducer and activator of transcription 1 (STAT1); hypoxia-inducible factor 1-alpha/Vascular endothelial growth factor A (HIF1A/VEGFA); Phosphatidylinositol-3 Kinase-AKT (PI3K-Akt); Vascular endothelial growth factor (VEGF); Transforming growth factor beta 1/Epithelial-mesenchymal transition (TGF-β1/EMT); NOD-like receptor family pyrin domain-containing 3 (NLRP3).

A significant elevation in the expression of circulating lncRNA NEAT1 was observed in individuals with T2DM, exhibiting a 5.28-fold increase compared to healthy subjects (158). LncRNA NEAT1 plays a crucial regulatory role in various diabetes-related complications, including diabetic retinopathy, diabetic nephropathy, and impaired wound healing associated with diabetes. The study by Yang et al. revealed that NEAT1 can modulate SOX4, thereby influencing the EMT in diabetic retinopathy through its interaction with miR-204. This finding offers a novel perspective and potentially identifies a valuable therapeutic target for addressing diabetic retinopathy (159). Furthermore, the study by Wang et al. demonstrates that lncRNA NEAT1 plays a significant role in the pathogenesis of diabetic nephropathy by modulating extracellular matrix proteins and EMT through the miR-27b-3p/ZEB1 axis (160). Complementary research has demonstrated the capacity of NEAT1 to promote diabetic nephropathy progression via activation of the Akt/mTOR signaling pathway. Considering these findings in conjunction with our results, NEAT1 holds significant potential as both a diagnostic biomarker and a therapeutic target in the management of diabetic nephropathy (161). The key lncRNAs in diabetic wound healing are systematically summarized (**Table 2**, **Figure 3**).

**Table 2:** Role of lncRNAs in the regulation of diabetic wound healing.

lncRNAs	Expressions	Animal models	Regulation mechanisms	References
lncRNA H19	Significantly decreased in diabetic wounds compared to normal wound	1) Mice were intraperitoneally injected with STZ solution (140 mg/kg, dissolved in 0.1 M sodium citrate buffer) and HF diet for an additional four weeks. Full-thickness excisional wound measuring 0.8 cm × 0.8 cm on the dorsal skin of each mouse 2) Male mice were intraperitoneally injected with 0.45% STZ. Full-thickness wounds (10 mm diameter) were made on the back 3) For inducing T2D mice were maintained on a high-fat diet for 12 weeks, and the dorsal skin at the midline of the mouse shoulder was excised for a full-thickness wound with a 6 mm biopsy punch 4) Mice were rendered diabetes by intraperitoneally injected of 0.45% STZ. 5) 0.45% STZ diluted by 0.1 mmol/L sterile sodium citrate buffer solution (45 mg/kg) was injected intraperitoneally to the rats. A mark (6 mm × 6 mm) was created on foot of rat with a sterile perforator, and the whole skin of the mark was excised 6) Adult male mice between 4 and 6 weeks old	1) Promotes the proliferation and migration of HaCaT cells by suppressing the activation of the NLRP3 inflammasome 2) Suppresses apoptosis, promotes the proliferation and migration of fibroblasts by interacting with miR-152-3p via PTEN-mediated PI3K/AKT signaling pathway 3) Promotes dermal fibroblast proliferation and macrophage infiltration by repressing fibroblast derived GDF15 4) Promotes the proliferation and migration of fibroblasts by binding miR-29b and upregulating FBN1. 5) Promotes fibroblast proliferation, ECM remodeling an angiogenesis by recruiting SRF and elevating CTGF levels 6) Promotes M2 polarization by targeting miR-130b-3p and regulating PPARγ/STAT	1) (145) 2) (206) 3) (146) 4) (148) 5) (147) 6) (133)
lncRNA GAS5	Downregulated in diabetic skin.	1) Diabetes was induced in mice by administering STZ (50 mg/kg) every other day. 2 Diabetes was induced by the intraperitoneal administration of STZ (45 mg/kg body weight in 0.1 M citrate buffer, pH 4.5) for five consecutive days. 3 mm × 4 mm wounds on the dorsal surface of the foot of each mouse 3) Genetically diabetic mice were given a full-thickness dorsal wound using an 8-mm punch biopsy	1) Promotes lymphangiogenesis by sponging miR-217 and up-regulating Prox1 2) Promotes HUVEC functions thereby enhances angiogenesis by binding to TAF15 and activating the HIF1A/VEGF 3) Inhibition of GAS5 promotes M2 polarization	1) (207) 2) (140) 3) (134)
MALAT1	Upregulated in diabetic mouse wounds compared to non-diabetic wounds	1) Healthy mice fed with a high-glucose and high-fat diet for 4 weeks and intraperitoneally injected with 0.45% STZ. Full-thickness wounds (10 mm diameter) 2) Genetically diabetic mice. A single dorsal full-thickness wound was made with an 8 mm punch biopsy	1) Promotes M2 polarization by suppressing miR-1914-3p to activate MFGE8 2) MALAT1 decreased TGF-β1-induced EMT in HaCaT cells.	1) (135) 2) (153)
lncRNA ANRIL	Downregulated in peripheral blood samples of DFU patients, and in skin tissues of DFU mice	Mice were administered STZ (45 mg/kg) intraperitoneally for 5 days. Full-thickness wounds (10 mm diameter) were made on the back of feet	Promotes angiogenesis by modulating HIF1A/VEGFA	(141)
lncRNA CASC2	Poorly expressed in wound tissues of DFU patients	Mice received an intraperitoneal injection of STZ (50 mg/kg). The skin of the mice feet (4 cm^2^) was removed	Promotes fibroblasts migration, proliferation, and inhibited apoptosis through miR-155/HIF-1α pathway	(152)
lncRNA XIST	Reduced in the skin tissues of rats with diabetic ulcers	Rats were injected with 1% STZ (40 mg/kg) after being fed a high-sugar and high-fat diet for one month	Promotes proliferation and migration of HaCaT cells miR-126-3p/EGFR pathway	(157)
lnc-URIDS	Upregulated in Diabetic Skin and Dermal Fibroblasts Treated With AGEs	Rats were intraperitoneally injected with 60 mg/kg STZ and allowed to manifest hyperglycemia for 4 weeks before making a cutaneous wound	Dysregulates collagen production by targeting PLOD1	(208)
lncRNA SNHG16	Increased in DFU tissue samples	N/A	Inhibition of lncRNA SNHG16 promotes proliferation and migration of HDF by Sponging miR-31-5p	(93)

## 5 Circular RNAs

CircRNAs, identified as key regulators of cell proliferation, metabolism, apoptosis, and inflammation, present as closed loops without poly A tails (162), establishing their significant involvement in diseases such as diabetes, cardiovascular disease, neurological disorders, and autoimmune disease (163–165). The primary roles of circRNA include serving as transcriptional regulators, functioning as miRNA sponges, acting as biomarkers for clinical diagnosis and treatment, and interacting with RNA-binding proteins (166–168).

Circ-Snhg11 has been identified to have regulatory effects at various stages of diabetic wound healing. Tang et al. demonstrated that circ-Snhg11 in BMSC-Exos improves angiogenesis and promotes diabetic wound healing by targeting miR-144-3p and enhancing SLC7A11/GPX4-mediated anti-ferroptosis signals (169). Furthermore, it was found that increasing levels of circ-Snhg11 counteracts the impaired function of EPCs and enhances M2 macrophage polarization under HG conditions. The study suggests that the upregulation of circ-Snhg11 inhibits ECs damage and promotes M2 macrophage polarization through the miR-144–3p/HIF-1α/VEGF pathway (170). Yin et al. demonstrated that circRps5, carried by ADSCs, promotes diabetic wound healing in diabetic mice by modulating macrophage polarization. Furthermore, miR-124-3p has been identified as a downstream target of circRps5. Thus, circRps5 delivered by ADSC-derived exosomes enhances M2 polarization, thereby facilitating diabetic wound healing through the regulation of miR-124-3p (171).

EPCs play a vital role in diabetic wound healing by proliferating and differentiating to form new blood vessels at sites of injury. However, a high glucose environment leads to EPC dysfunction, and multiple studies have found that circRNAs impact the regulation of EPC functions (172). The study by Shang et al. found that hypoxic pretreatment of EPCs promotes their survival and enhances diabetic wound healing by increasing autophagy through the overexpression of circ-Klhl8. MiR-212-3p was identified as the target of circ-Klhl8, and the downregulation of miR-212-3p promotes SIRT5 expression, thereby facilitating diabetic wound healing (173). *In vitro*, experiments have shown that exosomes derived from circ-Astn1-modified ADSCs play crucial roles in restoring the function of EPCs by reducing apoptosis under HG conditions. Furthermore, Wang et al. demonstrate that circ-Astn1 in ADSC-derived exosomes promotes diabetic wound healing by targeting miR-138-5p and upregulating SIRT1, which in turn decreases FOXO1 expression (174). mmu_circ_0000250 was also found to regulate EPC functions. Exosomes derived from mmu_circ_0000250-modified ADSCs plays a crucial role in restoring EPC function by activating autophagy, targeting miR-128-3p, and increasing SIRT1 levels (175). Tang et al. demonstrated that both exosomes from ADSCs and those from hypoxic pretreated ADSCs promote wound healing in diabetic mice, however, the exosomes from hypoxic pretreated ADSCs exhibit a greater therapeutic effect, with circ-Erbb2ip playing a significant role in this process. Bioinformatics analyses indicated that miR-670-5p is a target of circ-Erbb2ip, and further revealed that Nrf1 is a downstream target of miR-670-5p. These findings suggest that exosomes derived from hypoxic pretreated ADSCs enhance diabetic wound healing by delivering circ-Erbb2ip, which activates the miR-670-5p/Nrf1 signaling pathway. This pathway, in turn, reduces ROS levels and inflammatory cytokine expression while restoring the angiogenic function of EPCs (176).

Multiple studies have introduced the roles of circRNAs in the regulation of keratinocyte functions. Levels of circRNA-080968 were observed to be considerably elevated in DFUs samples relative to skin from diabetic patients without DFUs. Additionally, it was discovered that circRNA-080968 inversely affects the expressions of miR-326 and miR-766-3p by facilitating their breakdown, which in turn suppresses keratinocyte migration and promotes cell proliferation in DFUs (177). The levels of Circ_PRKDC at the wound edge were found to decrease significantly both 1 day and 7 days after the injury occurred. *In vitro* studies indicated that circ_PRKDC inhibits human epidermal keratinocyte (HEKs) migration by targeting and reducing miR-31 levels in keratinocytes, with FBN1 being a target of miR-31. Considering the role of FBN1 in promoting apoptosis and inhibiting fibroblast proliferation, it contributes to slower healing in DFU. Han et al. concluded that circ_PRKDC inhibition enhances wound healing by stimulating keratinocyte migration through the miR-31/FBN1 pathway (178). It was initially discovered that hsa_circ_0084443 expression was elevated in DFU, compared to normal wounds, and that this circRNA inhibits keratinocyte migration but promotes their proliferation through various gene regulatory networks (179). Huang et al. demonstrated that circCDK13 is a promising therapeutic agent for diabetic wound healing. They found that circCDK13 cooperates with IGF2BP3, and together they synergistically promote the proliferation and migration of human dermal fibroblasts and HEKs by enhancing the expression of c-MYC and CD44. Furthermore, to maximize the potential application of circCDK13 in wound healing and tissue regeneration, a delivery method was established. CircCDK13 loaded into small EVs promotes the proliferation and migration of human dermal fibroblasts and HEKs *in vitro* and accelerates diabetic wound healing in db/db diabetic mice (180).

Meng et al. demonstrated that by competitively binding hsa-miR-1273h-5p, hsa_circ_0008500 effectively neutralizes its ability to suppress ETS transcription factor ELK1 (ELK1) expression, playing a crucial role in inhibiting the apoptosis of ADSCs (181). Another study by Meng et al. investigated that circARHGAP12 promotes autophagy against MSC apoptosis under HG conditions by targeting miR-301b-3p and boosting the levels of autophagy-related 16-like 1 (ATG16L1) and unc-51 like autophagy activating kinase 2 (ULK2), thereby facilitating diabetic wound healing (182). Chen et al. demonstrated that exosomes overexpressing circ-ITCH promote wound healing in DFUs mice by alleviating ferroptosis and promoting angiogenesis in HG-treated HUVECs by recruiting TAF15 and activating the Nrf2 (183). Exosomal circHIPK3 has been identified as advantageous for healing diabetic wounds by enhancing angiogenesis through the miR-20b-5p/Nrf2/VEGFA pathway (184).

The study by Yang et al. initially discovered that delivering circ-Amotl1 enhances wound healing in mice. Furthermore, circ-Amotl1 was found to enhance wound healing by facilitating cell proliferation, survival, and migration through the enhanced nuclear translocation of Stat3. Stat3 then binds to the DNA methyltransferase 3A (Dnmt3a) promoter, reducing miR-17-5p expression but upregulating fibronectin levels. The decreased levels of miR-17-5p enhanced the expression of various proteins, which facilitates cell proliferation and migration, thereby accelerating wound repair (185). **Table 3** below highlights key circRNAs involved in diabetic wound healing.

**Table 3:** Role of circRNAs in the regulation of diabetic wound healing.

circRNAs	Expressions	Animal models	Regulation mechanisms	References
circ-Snhg11	Decreased in EPCs under HG conditions	1) Mice were injected an intraperitoneal injection regarding 60 mg/kg in STZ dissolved in 0.1 M citrate buffer, pH 4.5 2) Mice were injected with a single intraperitoneal injection of 60 mg/kg STZ dissolved in 0.1 M citrate buffer. Both had 4 mm full-thickness excisional wound	1) Promotes M2 polarization through miR-144–3p/HIF-1α/VEGF 2) Targets miR-144-3p and enhances SLC7A11/GPX4-mediated anti-ferroptosis signals and improves angiogenesis	1) (170) 2) (169)
circ-ITCH	Downregulated in *in vitro* and *in vivo* models of DFU	Mice were injected with STZ (45 mg/kg in 0.1 M citrate buffer, pH 4.5), and a square wound (1 × 1 cm) was created on the dorsal surface of the foot	Inhibits ferroptosis and improves angiogenesis of HUVECs by upregulating TAF15 and activating the Nrf2 pathway.	(183)
circ_0080968	Significantly higher in DFU samples compared to skins from non-DFU diabetic patients and normal human wounds	N/A	Inhibits wound healing by targeting miR-326 and miR-766-3p, which further represses the migration and increases the proliferation of keratinocytes	(177)
hsa_circ_0084443	Upregulated in DFU compared to normal wounds	N/A	Negatively regulates keratinocyte migration	(179)
circ-Klhl8	Hypoxic pretreatment promoted circ-Klhl8 expression in EPCs	Mice were injected with STZ (60 mg/kg in 0.1 M citrate buffer, pH 4.5), and a 4 mm full-thickness excisional wound was created on the dorsal skin	Increases the EPC therapeutic effect by targeting miR-212-3p and promoting SIRT5 expression	(173)
circARHGAP12	Reduction in MSCs treated with HG	Mice with Type 1 diabetes had skin wounds of 1.2 cm in diameter.	Enhances MSC autophagy to protect MSCs against apoptosis by promoting the expression of ATG16L1 and ULK2 by targeting miR-301b-3p	(182)
circ-Astn1	Increased in ADSC exosomes compared with exosomes from fibroblasts	Diabetes was induced in mice through a single intraperitoneal injection of 60 mg/kg STZ dissolved in 0.1 M citrate buffer (pH 4.5), and a 4 mm full-thickness excisional wound was created on the dorsal skin	Restoring the function of EPCs by reducing apoptosis under HG conditions	(174)
circHIPK3	Upregulated in type 2 diabetes mellitus	Diabetes was induced in mice through an injection of 100 mg/mL STZ into mice at 200 mg/kg	Promotes Angiogenesis of HUVECs by targeting miR-20b-5p and upregulating Nrf2 and VEGFA	(184)
hsa_circ_0008500	Downregulated under high glucose	N/A	Suppresses apoptosis in ADSCs by targeting hsa-miR-1273h-5p to neutralize its ability to suppress ELK1expression	(163)
circ_PRKDC	Downregulated 1.2-fold 1 day (the inflammatory phase) and 3.4-fold 7 days post wounding (the proliferative phase)	N/A	Inhibition of circ_PRKDC promotes keratinocyte migration through miR-31/FBN1 pathway	(178)

## 6 Limitations and perspectives

This article has reviewed the roles of different classes of ncRNAs, including miRNAs, lncRNAs, and circRNAs, in diabetic wound healing. The detailed examination of these ncRNAs highlights their impact on the regulation of critical processes such as inflammation, angiogenesis, re-epithelialization, and extracellular matrix remodeling, which are important for effective wound repair, particularly in diabetes conditions, where the healing process is widely slow and often incomplete. While most current research studies have investigated the potential mechanisms by which ncRNAs influence diabetic wound healing, several limitations in these studies necessitate further investigation.

Current research mostly focuses on miRNAs, with relatively less attention given to circRNAs, lncRNAs, and other types of ncRNAs. This imbalance might limit our understanding of unique biological functions that these lesser studied ncRNAs could have in wound healing for diagnosis and clinical applications.

Similarly, while much of the existing research has concentrated on fibroblasts, keratinocytes, and ECs, there is a need for more comprehensive studies on the role of immune cells. Immune cells like lymphocytes, dendritic cells, and others significantly influence healing outcomes by being involved in almost all key stages of wound healing and it is important to coordinate the activities of immune cells accurately (186). The imbalance of immune cells can result in the degradation of the immune microenvironment (187). A detailed exploration of ncRNAs’ roles in these immune cells could unveil new mechanisms of immune regulation in diabetic wounds.

Another gap in current studies is the emphasis on the final effect without exploring their specific roles in each phase of wound healing. This gap leaves an incomplete understanding of the temporal dynamics of ncRNAs action during the critical stages of inflammation, proliferation, and remodeling. Conducting phase-specific research could help identify new therapies that target these specific phases, leading to optimized healing processes.

Finally, there is a lack of studies examining the complex interactions among ECs, fibroblasts, keratinocytes, and immune cells in the context of ncRNAs mediation. Understanding how ncRNAs facilitate or impair cell-cell communication and coordination in wound environments could offer valuable insights into the mechanisms of wound healing. Despite these limitations, the research into ncRNAs offers considerable potential for improving the treatment of DFUs. Here we outline several future perspectives that not only address these challenges but also lay the foundation for major advancements in this field.

The application of ncRNAs as biomarkers for diabetic wound healing offers a promising frontier for early diagnosis and prognosis. Prospective studies designed to validate specific ncRNAs as reliable biomarkers could lead to the development of predictive tools for wound healing success or failure. Among ncRNAs, circRNAs are particularly promising due to their unique closed-loop structure, which, unlike linear ncRNAs, provides enhanced stability and resistance to exonucleases (188). Future studies should not only validate ncRNAs in retrospective settings but also prospective analyses. This approach could accelerate the research process, enabling quicker integration of ncRNA biomarkers into clinical practice, thereby enhancing diagnostic accuracy and patient management strategies.

Beyond their potential as biomarkers, ncRNAs also hold promise as therapeutic agents in diabetic wound healing. While current treatments for diabetic wound healing exist, ncRNA-based therapies hold promise for more precisely targeted intervention strategies. By selectively regulating gene expression, ncRNAs can potentially accelerate wound repair and reduce the risk of complications, offering an innovative approach to diabetic wound care.

Recent advancements in nanomedicine have greatly improved the stability, targeting precision, and therapeutic efficacy of exogenous ncRNAs in wound healing. Nanoparticle-based delivery systems, such as lipid nanoparticles and polymer-based carriers, protect ncRNAs from enzymatic degradation, enhancing their stability, bioavailability, and longevity in the bloodstream (189). Functionalized nanoparticles with specific ligands improve targeting efficiency by directing ncRNAs to wound-specific cells like fibroblasts and keratinocytes, thereby enhancing therapeutic outcomes. Additionally, engineered nanoparticles provide controlled, sustained release of ncRNAs, enabling precise regulation of molecular pathways involved in inflammation, angiogenesis, and tissue repair (190). Addressing off-target effects remains a critical consideration in the development of effective miRNA-based therapies, and these recent advances not only accelerate wound healing but also address challenges like off-target effects and long-term safety concerns. Ongoing research is focused on improving nanoparticle biocompatibility and developing condition-responsive systems for more precise ncRNA delivery in clinical applications (191).

While delivering ncRNAs through various pathways can lead to both upregulation and downregulation of ncRNA expression in diabetic wounds, the regulation of endogenous ncRNAs is highly complex and influenced by various metabolic abnormalities. Hyperglycemia in diabetic patients causes an overproduction of ROS, leading to oxidative stress (19, 192). This condition significantly impacts the expression of ncRNAs, particularly those involved in inflammatory responses and tissue repair. For example, in diabetic wounds, hyperglycemia-induced oxidative stress triggers an unregulated and prolonged unfolded protein response (UPR), resulting in a deficiency of inositol-requiring enzyme 1 (IRE1α), which is a key transducer of the UPR that regulates the expression of mRNAs and miRNAs. This deficiency causes an increase in the levels of the miR-200 family and miR-466 (193). Furthermore, inflammatory cytokines such as TNF-α and IL-6 also influence ncRNA expression, further complicating the regulatory processes (194). In addition to metabolic factors, infections are another significant factor that can regulate the expression of endogenous ncRNAs. The presence of pathogens can induce inflammatory responses, leading to alterations in ncRNA expression patterns (195, 196).

However, to turn this potential into reality, more clinical trials are needed. The limited number of clinical trials testing ncRNAs therapies underscores a significant gap in extensive clinical investigation. To advance the field, future research should focus on translating preclinical findings into clinical trials that evaluate the therapeutic efficacy and safety of ncRNA-based interventions. These trials should be comprehensive, involving multi-center collaborations to ensure a diverse patient cohort, with long-term follow-ups to assess both the safety and effectiveness of these therapies. By addressing these aspects, clinical trials can bridge the gap between experimental research and real-world medical applications.

## 7 Conclusion

The significance of ncRNAs in the diabetic wound healing process cannot be overstated. Through our comprehensive review, we have underscored the importance of miRNAs, lncRNAs, and circRNAs in diabetic wound healing. NcRNAs serve as crucial regulators, modulating critical aspects, such as inflammation, angiogenesis, re-epithelialization, and ECM remodeling. Our analysis highlights the impact of ncRNAs on the activities of fibroblasts, ECs, and keratinocytes, which are central players in wound healing through regulating protein interactions and signaling pathways.

The integration of ncRNAs into clinical practice holds immense promise, leveraging their diagnostic and therapeutic potential. The diagnostic potential of ncRNAs as biomarkers for diabetic wounds and related conditions offers early detection and personalized treatment options. Furthermore, exploring the therapeutic potential by silencing or activating ncRNAs could lead to novel interventions that enhance diabetic wound healing outcomes.

## Glossary

(ECM): Extracellular matrix
(FGF): fibroblast growth factors
(ROS): reactive oxygen species
(ECs): endothelial cells
(VEGF): vascular endothelial growth factor
(VEGFA): Vascular Endothelial Growth Factor A
(DFUs): diabetic foot ulcers
(IDF): International Diabetes Federation
(ncRNAs): non-coding RNAs
(miRNAs): including microRNAs
(lncRNAs): long non-coding RNAs
(circRNAs): circular RNAs
(piRNAs): piwi-interacting RNAs
(snoRNAs): small nucleolar RNAs
(3’-UTR): three prime untranslated regions
(mRNAs): messenger RNAs
(DM): diabetes mellitus
(T2DM): Type 2 diabetes mellitus
(NETs): neutrophil extracellular traps
(HUVECs): Human Umbilical Vein endothelial cells
(GDF6): targeting growth differentiation factor 6
(NF-κB): nuclear factor-kappa B
(IRAK1): interleukin-1 receptor-associated kinase 1
(CNP): cerium oxide nanoparticles
(SFP): silk fibroin patch
(TNF-α): Tumor Necrosis Factor-Alpha
(IL-1β): Interleukin-1 beta
(IL-6): Interleukin-6
(TRAF6): tumor necrosis factor receptor-associated factor 6
(PAK7): p21-Activated Kinase 7
(EVs): extracellular vesicles
(CDKN1A): cyclin-dependent kinase inhibitor 1A
(PDGFD): platelet-derived growth factor D
(ICAM-1): intercellular adhesion molecule 1
(LPS): lipopolysaccharide
(PTEN): phosphatase and tensin homolog
(PDCD4): programmed cell death 4
(DYRK1A): dual-specificity tyrosine-phosphorylation-regulated kinase 1A
(hTERT): human telomerase reverse transcriptase
(IκB): inhibitor of NF-kappa-B
(SOCS3): suppressor of cytokine signaling 3
(EGF): epidermal growth factor
(HIP1): huntingtin interacting protein 1
(IGF2): insulin-like growth factor 2
(RASSF2): Ras association domain-containing protein 2
(ROCK1): Rho-associated protein kinase 1
(CITED2): Cbp/p300-interacting transactivator 2
(HIF-1): hypoxia-inducible factor 1
(HIPK2): homeodomain-interacting protein kinase 2
(THBS1): thrombospondin 1
(NOTCH1): neurogenic locus notch homolog protein 1
(HIF1AN): hypoxia-inducible factor 1-alpha inhibitor
(STARD13): StAR-related lipid transfer protein 13
(SPARC): secreted protein acidic and rich in cysteine
(SDF-1α): stromal cell-derived factor 1-alpha
(PIK3R3): phosphoinositide-3-kinase regulatory subunit 3
(CYP1B1): cytochrome P450 1B1
(MeCP2): methyl-CpG-binding protein 2
(STZ): streptozotocin
(db/db): diabetic mice
(Flk-1): fetal liver kinase 1
(IGF1R): insulin-like growth factor 1 receptor
(NFIC): nuclear factor I/C
(MAPKAPK2): mitogen-activated protein kinase-activated protein kinase 2
(MMP1): matrix metalloproteinase-1
(FN1): fibronectin 1
(SPRY1): sprouty homolog 1
(NOVA1): neuro-oncological ventral antigen 1
(MMP-9): matrix metalloproteinase-9
(Sp1): specificity protein 1
(ADSC): adipose-derived stem cells
(HG): high glucose
(MFGE8): milk fat globule-EGF factor 8 protein
(NOX2): NADPH oxidase 2
(EMNVs): extracellular vesicle-mimetic nanovesicles
(Es): endothelial progenitor cells
(TAF15): TATA-binding protein-associated factor 15
(NLRP3): NOD-like receptor family pyrin domain-containing 3
(GDF15): growth differentiation factor 15
(APCs): antigen-presenting cells
(CTGF): connective tissue growth factor
(FBN1): fibrillin-
(Smad3): Mothers against decapentaplegic homolog 3
(TGF-β1): transforming growth factor beta 1
(FN): fibronectin
(SOX9): SRY-related high-mobility-group box 9
(EZH2): enhancer of zeste homolog 2
(c-Myc): transcription factor
(EMT): epithelial-mesenchymal transition
(ZEB1): Zinc Finger E-box Binding Homeobox 1
(Nrf2): nuclear factor erythroid 2-related factor 2
(ZNF148): zinc finger protein 148
(ELK1): ETS transcription factor ELK1
(FBN): fibrillin
(ELK1): ETS transcription factor ELK1
(ATG16L1): autophagy-related 16-like 1
(ULK2): unc-51 like autophagy activating kinase 2
(Dnmt3a): DNA methyltransferase 3A
(HEKs): human epidermal keratinocyte
(UPR): unfolded protein response
(IRE1α): inositol-requiring enzyme 1
